# *Sasa quelpaertensis* leaf extract regulates microbial dysbiosis by modulating the composition and diversity of the microbiota in dextran sulfate sodium-induced colitis mice

**DOI:** 10.1186/s12906-016-1456-7

**Published:** 2016-11-25

**Authors:** Yiseul Yeom, Bong-Soo Kim, Se-Jae Kim, Yuri Kim

**Affiliations:** 1 Department of Nutritional Science and Food Management, Ewha Womans University, Seoul, 03760 Republic of Korea; 2 Department of Life Science, Hallym University, Chuncheon, Gangwon-do 24252 Republic of Korea; 3 Department of Biology, Jeju National University, Jejusi, Jeju 63243 Republic of Korea

**Keywords:** *Sasa quelpaertensis* leaf extract, Inflammatory bowel disease, Dextran sulfate sodium, Gut microbiota

## Abstract

**Background:**

Inflammatory bowel diseases (IBD) are related to a dysfunction of the mucosal immune system and they result from complex interactions between genetics and environmental factors, including lifestyle, diet, and the gut microbiome. Therefore, the effect of *Sasa quelpaertensis* leaf extract (SQE) on gut microbiota in a dextran sulfate sodium (DSS)-induced colitis mouse model was investigated with pyrosequencing of fecal samples.

**Methods:**

Three groups of animals were examined: *i*) a control group, *ii*) a group that was received 2.5% DSS in their drinking water for 7 days, followed by 7 days of untreated water, and then another 7 days of 2.5% DSS in their drinking water, and *iii*) a group that was presupplemented with SQE (300 mg/kg body weight) by gavage for two weeks prior to the same DSS treatment schedule described in *ii*.

**Results:**

SQE supplementation alleviated disease activity scores and shortened colon length compared to the other two groups. In the DSS group, the proportion of *Bacteroidetes* increased, whereas that the proportion of *Firmicutes* was decreased compared to the control group. SQE supplementation recovered the proportions of *Firmicutes* and *Bacteroidetes* back to control levels. Moreover, the diversity of microbiota in the SQE supplementation group higher than that of the DSS group.

**Conclusion:**

SQE was found to protect mice from microbial dysbiosis associated with colitis by modulating the microbial composition and diversity of the microbiota present. These results provide valuable insight into microbiota-food component interactions in IBD.

## Background

Inflammatory bowel diseases (IBD), including Crohn’s disease (CD) and ulcerative colitis (UC), are chronic and recurrent inflammatory disorders with uncertain etiology [1]. IBD are generally accompanied by abdominal pain, weight loss, and diarrhea, and can result in complete obstruction of the gastrointestinal (GI) tract [2]. Worldwide, the incidence and prevalence of IBD have increased over the past few decades. To date, the precise pathogenesis of IBD remains unclear, although dysfunction of the immune system due to interactions between a host’s response to microbial flora in the gut may be one of the main factors that contribute to these diseases [3].

Hunan gut microbiota consists of more than 100 trillion microorganisms, which is ten times more than the total number of human cells in the body [4]. In general, a fetus grows in a sterile environment in the uterus. Then, after birth, gut colonization starts rapidly and it is influenced by a variety of factors, including diet, antibiotics, and stress [5]. Gut microbiota have diverse and useful functions in energy balance, glucose metabolism, drug metabolism, and inflammation in a host [6]. However, when an imbalance in normal gut microbiota occurs, this is known as dysbiosis. Dysbiosis underlies the pathogenesis of numerous diseases, including IBD, colorectal cancer, and metabolic syndrome in connection with host metabolism [7, 8]. For obese individuals, their intestinal microbiota contains a higher proportion of *Firmicutes*, and a lower proportion of *Bacteriodetes* compared to lean individuals [9, 10]. Insulin sensitivity and plaque synthesis in blood vessels can also be altered by gut microbiota [9, 11]. Furthermore, changes in the population and metabolism of the diverse bacteria population in a GI tract can affect systemic inflammation and the function of neurotransmitters in the brain [12, 13].

Gut microbiota plays a critical role in anti-inflammatory and immune-regulatory function, and thus, potentially represent an attractive IBD therapy. Various therapies that target restoration of the gut microbiota by altering their composition have been suggested, including fecal microbiota transplantation, probiotics, prebiotics, antibiotics, and dietary intervention. Recent interest in the dietary phytonutrients that are present in natural herbs has led to investigations of their potential impact on human health. For example, the polyphenols present in various natural herbs have been reported to modulate the composition and numbers of gut microbiota and to indirectly influence metabolism and the bioavailability of gut microbiota [14]. Another key benefit that has been found is an absence of undesirable side effects. Thus, gut microbiota may represent a potential therapeutic strategy for IBD and may help maintain intestinal function [15].

*Sasa quelpaertensis* Nakai is an edible dwarf bamboo grass that inhabits the area surrounding Mt Halla on Jeju Island in Korea. Its leaf extract has been reported to mediate various health promoting properties, including anti-inflammation, anti-cancer effects, and anti-obesity effect [16–18]. *Sasa quelpaertensis* leaves extract (SQE) is a mixture of polysaccharides, amino acids, and polyphenols, including *p*-coumaric acid and tricin, and has exhibited anti-inflammatory and anti-obesity effects [19, 20]. In particular, SQE has been found to mediate anti-inflammatory effects by regulating inflammatory mediators such as nitric oxide, tumor necrosis factor α, and COX-2 both in vivo and in vitro [17]. However, there is limited evidence regarding the effect of SQE on gut microbiota during inflammation.

Therefore, in the present study, the ability of SQE to regulate inflammation by modulating microbial composition in a dextran sulfate sodium (DSS)-induced colitis animal model was evaluated using high-throughput sequencing of the 16S ribosomal rRNA (rRNA) gene.

## Methods

### Preparation of SQE

SQE was prepared as previously described [17]. *Sasa quelpaertensis* Nakai voucher specimen has been deposited in a publicly available herbarium name as HALLA ARBORETUM HERBARIUM and deposit number is HA006630. Briefly, collected *Sasa quelpaertensis* Nakai leaves (1 kg) were collected from Mt. Halla on Jeju Island, South Korea and were washed twice with deionized water. The leaves were then dried and extracted with 70% ethanol for 48 h at room temperature. After the SQE was filtered, it was concentrated with a rotary evaporator under reduced pressure and freeze-dried. The resulting SQE extract was crushed into a powder and stored at - 20 °C until needed. Previously, we have reported that *p*-coumaric acid and tricin were two major bioactive compounds in SQE and determined the concentrations of these compounds using high performance liquid chromatography (HPLC) 2695 Alliance System (Waters Corp., Mildford, MA, USA). The concentrations of each *p*-coumaric acid and tricin were 1.13 and 0.82 mg/g [17].

### Induction of DSS-induced colitis in mice

Five-week-old male C57BL/6 mice were purchased (Central Lab, Animal Inc., Seoul, Korea) and maintained under standard laboratory conditions: 22 ± 2 °C, 50 ± 5% humidity, and a 12 h/12 h light/dark cycles. Animals received a modified American Institute of Nutrition (AIN)-93G pellet diet (Unifaith, Inc., Seoul, Korea). Diet composition was provided in Table 1. To confirm their health status, all mice were housed for 1 week before being randomized into three groups (*n* = 6/group).

**Table 1:** Dietary composition for the experiment

Ingredients (g)	g/kg diet
Casein, lactic	200
L-cystein	3
Corn starch	397.5
Maltodextrin	132
Sucrose	100
Cellulose	50
Soybean Oil	70
Mineral mix, AIN-93G^a)^	35
Vitamin mix, AIN-93G	10
Cholin Bitartrate	2.5
t-butylhydroquinone	0.014

^a)^Mineral mixture and vitamin mixture were prepared according to AIN-93G diet

The three experimental groups included: i) mice receiving a standard diet and normal drinking water (control), ii) mice receiving 2.5% DSS (DSS), and iii) mice receiving DSS + SQE [300 mg/kg body weight (b.w.)] (SQE). The DSS and SQE groups received 2.5% DSS (molecular weight: 36 – 50 kDa; MP Biomedicals, Costa Mesa, CA, USA) in their drinking water for 7 d, followed by 7 d of untreated drinking water, and then another 7 d of 2.5% DSS in their drinking water. The SQE group mice received a daily oral dose of SQE for 14 d prior to DSS treatment. During the experimental period, body weight and diet intake were recorded twice a week. After five weeks, all of the mice were sacrificed. Animal care and experimental protocols for this study were approved by the Animal Care and Use Committee of Ewha Womans University (IACUC approval no: IACUC 14-070).

### Disease activity index (DAI)

DAI scoring was measured from the start of DSS administration until the end of the experimental period as described previously [17]. DAI scores were determined based on weight loss, stool consistency, and fecal bleeding. Stool consistency was evaluated according to the presence of loose feces and watery diarrhea. Fecal bleeding was scored as normal, slightly bloody, and blood in whole colon compared to the control group.

### Genomic DNA extraction

To analyze gut microbiota analysis, fecal samples were collected at 19 d after the start of the DSS treatment. Metagenomic DNA was extracted with Fast DNA SPIN Kits (MP BIO, Santa Ana, CA, USA), according to the manufacturer’s instructions. The resulting metagenomic DNA samples were dissolved in 50 μl of elution buffer and stored at - 20 °C until needed. DNA concentrations were determined based on optimal density value obtained at 260 nm. Sample purity was determined based on the ratio of the absorbance values obtained at 260 nm and 280 nm.

### Pyrosequencing analysis of gut microbiota based on the 16S rRNA gene

The 16S rRNA gene (targeted V1-V3 regions) was amplified from the extracted DNA using barcoded primers (27 F and 518R). The resulting PCR products were confirmed by gel electrophoresis and purified. Sequencing of the amplicons was conducted using a Roche/454 GS Junior system (ChunLab, Inc., Seoul, Korea). Data analysis was performed according to previously described method [21]. Each sample was sorted according to a unique barcode. Low quality reads (average quality score < 25 or read length < 300 bp) did not undergo further analysis. The primer sequences were trimmed and clustered for correcting sequencing errors. The taxonomic positions of the representative sequences for each cluster were identified using the EzTaxon-e database [22]. Chimeric sequences were removed using the UCHIME program [23] and the diversity indices were calculated with the Mothur program [24]. The pyrosequences presented in this study are available in the EMBL SRA database under the study PRJEB13815 (http://www.ebi.ac.uk/ena/data/view/PRJEB13815). The operational taxonomic unit (OTUs) were mathematically defined as having a 3% sequence distance (e.g. 97% similarity). Diversity and richness were calculated using the Cluster Database at High Identity with Tolerance (CD-HIT). Alpha diversity indices such as Chao1 and Shannon diversity were used to estimate species richness using the Mothur program and the matrix of Fast UniFrac. Principal coordinate analysis (PCoA) was used to represent the relationships between samples based on calculations of Jaccard abundance similarity and Bray-Curtis similarity [24, 25].

### Statistical analysis

Statistical analyses were performed using GraphPad PRISM software (GraphPad Software, SanDiego, CA, USA). Data presented are the mean ± standard error of the mean (SEM) for each group. For multiple comparisons, one-way analysis of variance (ANOVA) with Newman-Keuls’s post-hoc test was used. A *P*-value less than 0.05 was considered statistically significant.

## Result

### DAI and colon length

**Fig. 1:**
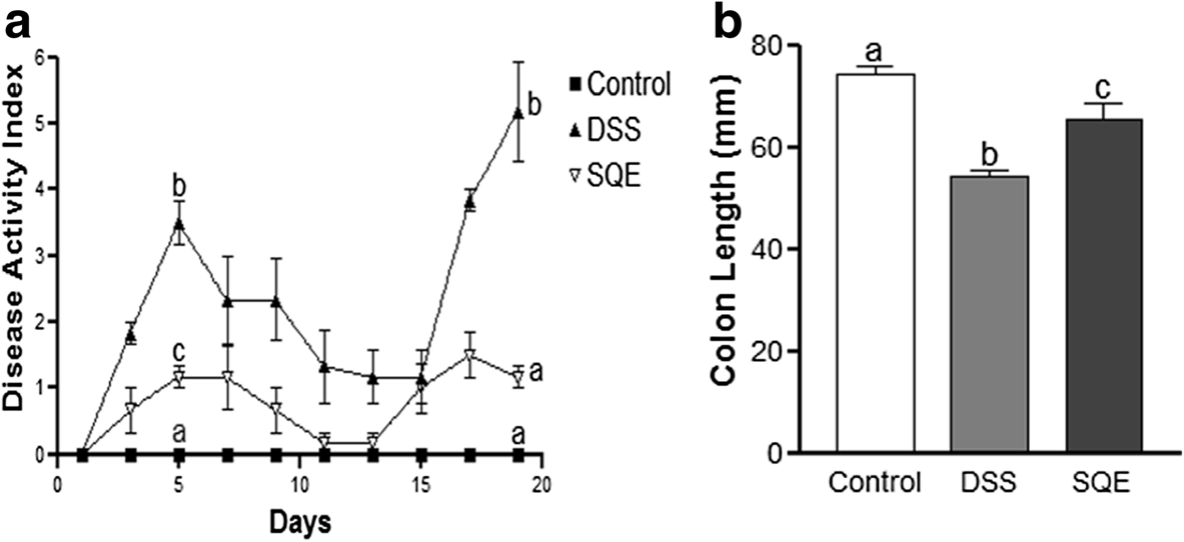
DAI scores and colon length in the DSS-induced colitis. **a** DAI values were evaluated based on observed changes and scoring of body weight loss, stool consistency, and fecal bleeding. **b**, Colon length was measured and compared among the control, DSS, and SQE groups. Data shown are the means ± SEM and were analyzed by one-way ANOVA and Newman-Keuls’s post hoc test (*p* < 0.05); *n* = 6 mice per group

DAI scores were significantly increased up to day 5 and peaked on day 19 in the DSS group compared to the control group (Fig. 1a). The increase in DAI values was based on the observed incidence of diarrhea, weight loss, and bloody stools. In contrast, the DAI scores of the SQE group were significantly attenuated by 61.9% at day 5 and by 77.4% at day 19 compared to the DSS group (*p* < 0.05 in each case). Moreover, the DAI score for the SQE group was comparable to that of the control group.

Since severity of DSS-induced colitis was found to be associated with a shorter colon length [26], whole colon tissues were isolated from each group and their lengths were and compared. The mean colon length of the DSS group was 27% shorter than the mean colon length of the control group, and SQE supplementation significantly attenuated shortening of the colon compared with the DSS group (Fig. 1b).

### OTUs and diversity estimates for fecal microbiota

The average numbers of analyzed sequence reads were 5310 ± 1519 for the control group and 4722 ± 1092 for the DSS group, and 4744 ± 1092 for the SQE group (Table 2). The Good’s coverages of all the samples were greater than 0.97. The number of observed OTUs was 444.83 ± 66.84 for the control group, 256.33 ± 65.64 for the DSS group, and 404.17 ± 178.21 for the SQE group. The number of observed OTUs was significantly lower for the DSS group compared with the control group by 42.4%, while the number of OTUs in the SQE group tended to be greater than the number of OTUs for the DSS group (57.7%). Similarly, the number of estimated OTUs (Chao1) in the DSS group was significantly lower than those for the control group (*p* < 0.05, 44.3%), while those for the SQE group were higher compared to the DSS group (*p* < 0.05, 62.2%). The Shannon diversity indices for the control group were also significantly higher than those for the DSS group, yet were similar to those of the SQE group. Taken together, these results indicated that the diversity of gut microbiota in the DSS group were more diverse than the gut microbiota of the control and SQE groups.

**Table 2:** Summary of diversity indices obtained from pyrosequencing results

	Control	DSS	SQE
Analyzed sequence reads (avg.)	5310 ± 1519	4722 ± 1092	4744 ± 1096
Goods Coverage	0.97 ± 0.01	0.98 ± 0.01	0.97 ± 0.01
Observed OTUs	444.83 ± 66.84 ^a^	256.33 ± 65.64 ^b^	404.17 ± 178.21 ^ab^
Chao1 estimators	657.94 ± 91.98 ^a^	366.26 ± 109.76 ^b^	594.05 ± 259.98 ^a^
Shannon diversity index	4.75 ± 0.19 ^a^	3.96 ± 0.31^b^	4.52 ± 0.69 ^a^

Values are mean ± SD

Significantly different by one – way ANOVA and Newman-Keuls’s post hoc test among the three groups (*p* < 0.05); *n* = 6 mice per group.

^abc^For a given column, data not sharing a common superscript letter significantly differ

### Comparison of gut microbiota

**Fig. 2:**
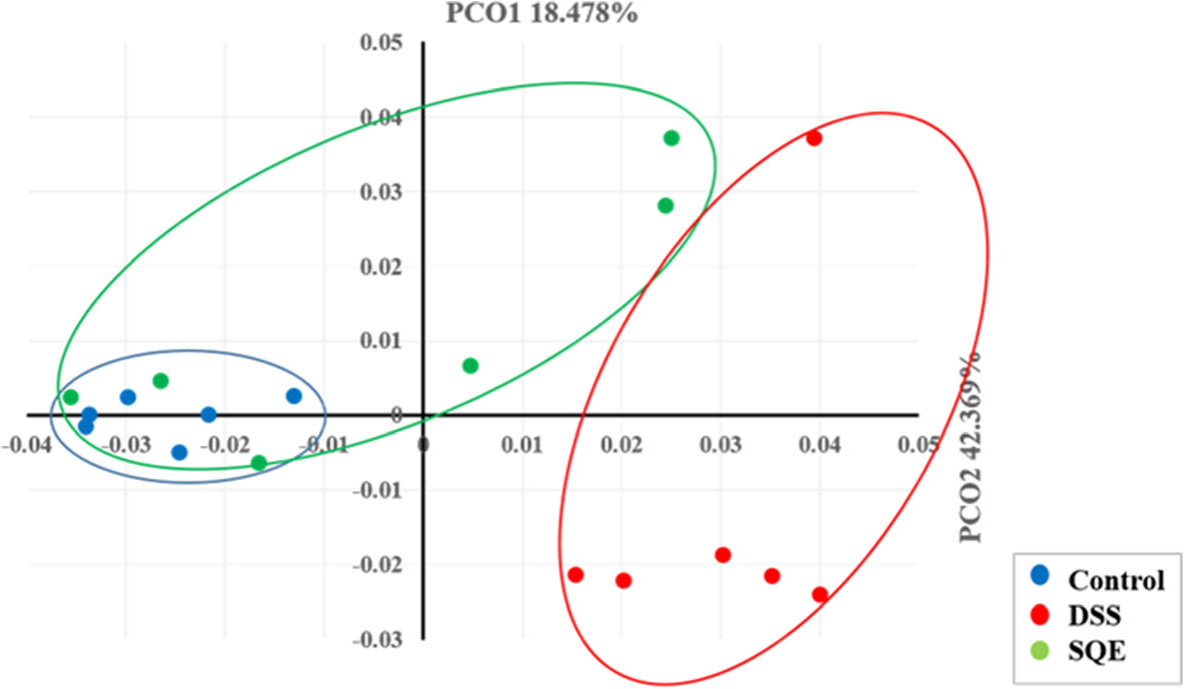
Principal coordinate analysis (PCoA) plot. PCoA was used to determine clustering patterns among the control, DSS, and SQE groups (*n* = 6 mice/group). Similarities between the communities were calculated by employing Fast UniFrac analysis

To compare microbial community members among the three groups, clustering patterns based on a weighed pairwise Fast UniFrac analysis was determined (Fig. 2). The gut microbiota obtained from the DSS group was distinct from those of the control and SQE group, and the gut microbiota of the SQE group were closer to the control group in PCoA plot.

### Comparison of gut microbiota composition with pyrosequencing

**Fig. 3:**
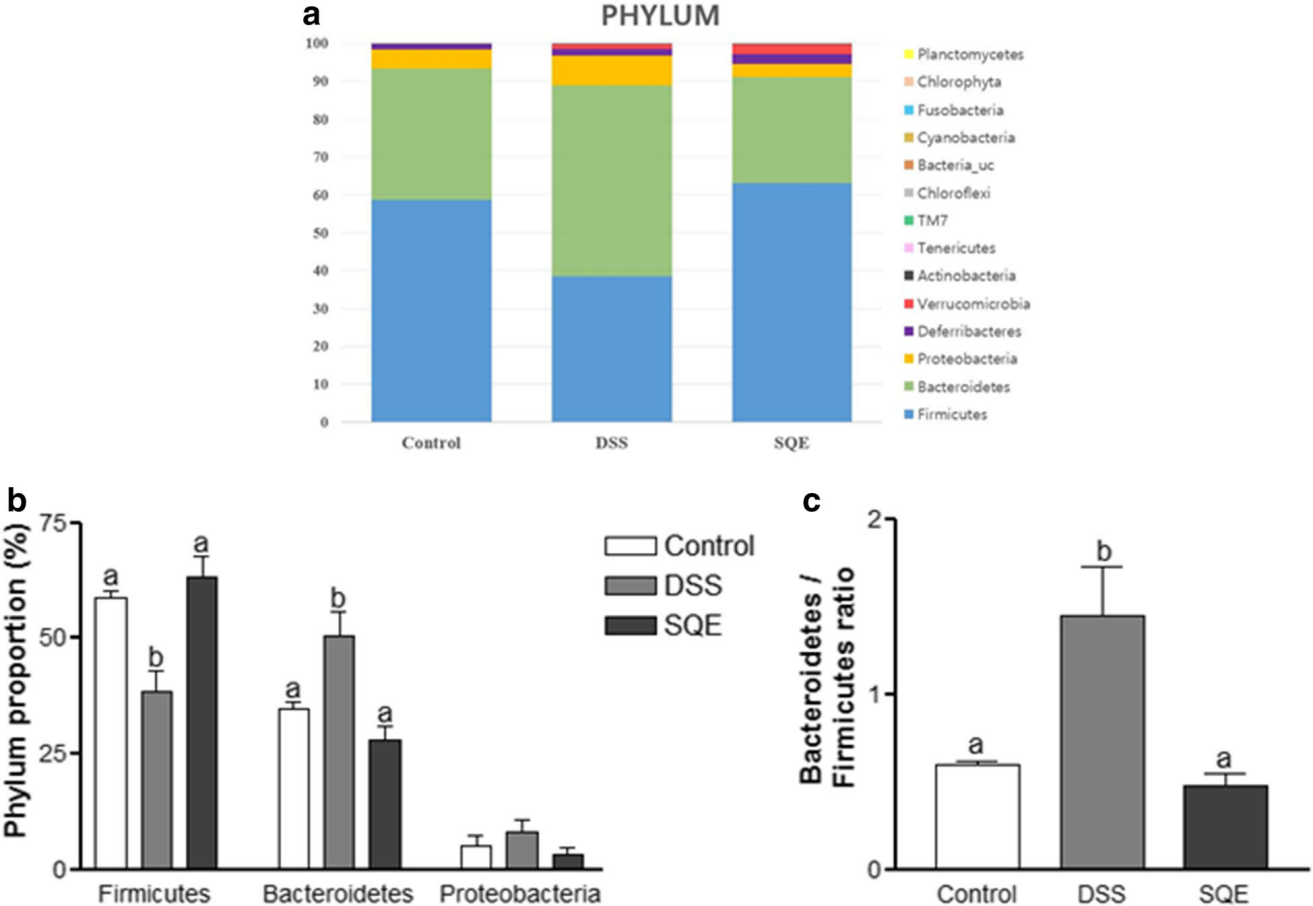
Composition of the gut microbiota at the phylum level. **a** The composition of gut microbiota at the phylum level. **b**, **c** Changes in the proportion of major class (**b**) and minor class (**c**) bacterial at the phylum level among the control, DSS, and SQE groups. **c** Relative abundance of phylum level of minor proportion of bacteria in control, DSS, and SQE group. Data shown are the means ± SEM and were analyzed by one-way ANOVA and Newman-Keuls’s post hoc test (*p* < 0.05); *n* = 6 mice per group

To analyze gut microbiota, fecal samples were collected at day 19 d after the start of the DSS treatment and they were analyzed using pyrosequencing. Differences in the microbiota among the groups were compared at the phylum level (Fig. 3). DSS treatment greatly increased the levels of *Bacteroidetes* by 44.9%, and decreased the levels of *Firmicutes* by 34.4% compared to the control group (Fig. 3a and b). Correspondingly, the ratio of *Bacteroidetes* to *Firmicutes* in the gut microbiota was higher for the DSS group compared to the control group (Fig. 3c). However, following SQE supplementation, the proportions of *Bacteroidetes* and *Firmicutes* returned to control levels. Moreover, the ratio of *Bacteroidetes* to *Firmicutes* decreased following SQE supplementation. In contrast, the levels of *Proteobacteria* and *Deferribacteres* did not significantly different among the three groups.

**Fig. 4:**
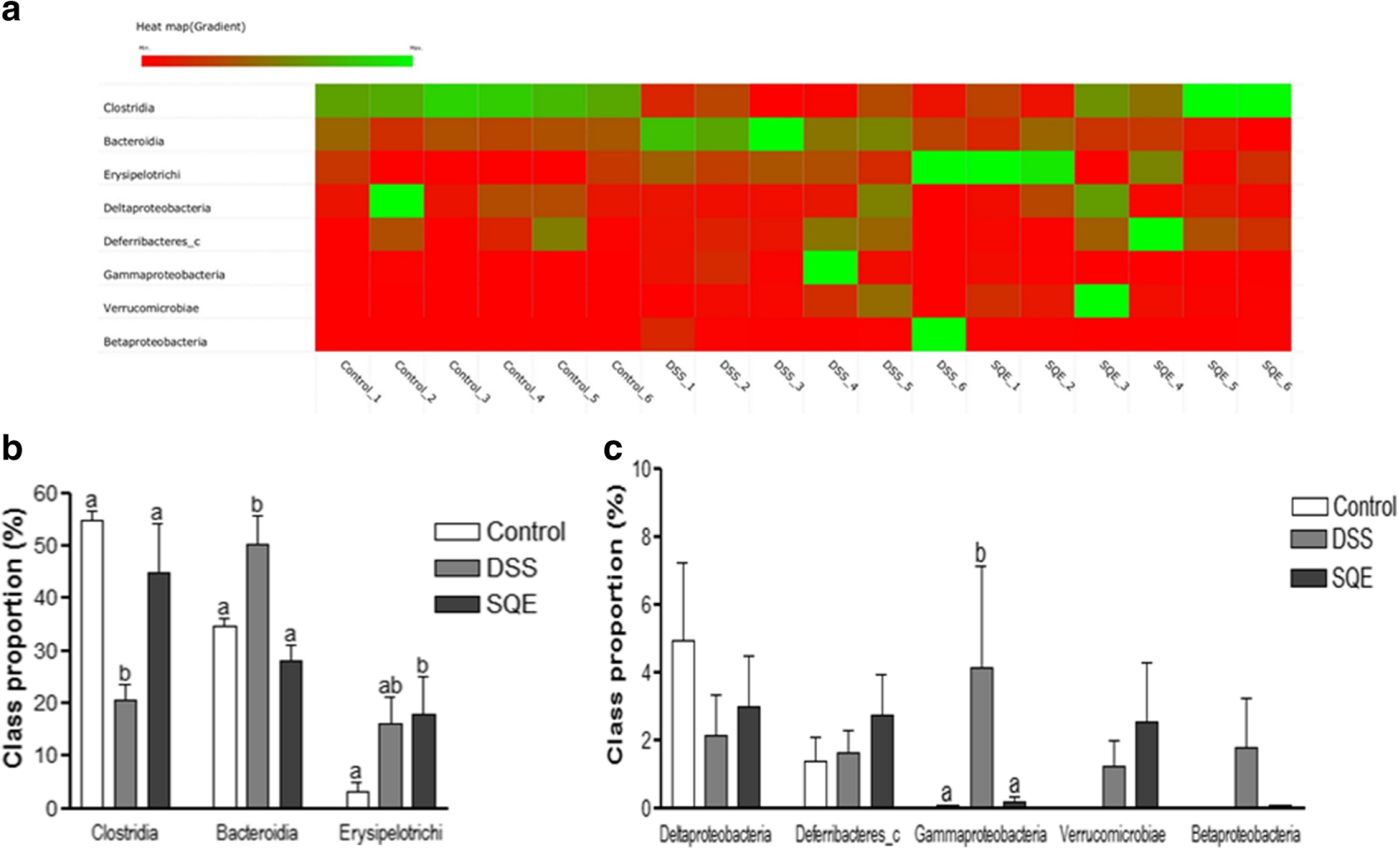
Taxonomy composition of the gut microbiota at the class level. **a** A heatmap analysis of the class levels for the three experimental groups. Genomic DNA was extracted from the fecal samples obtained 19 d after the start of DSS treatment. The samples were analyzed for their bacterial composition based on pyrosequencing of 16S rRNA. The data are represented by red and green colors and the cut-off value was set at 5% (**b**, **c**) Relative abundance of the major gut microbiota at the class levels. Data shown are the means ± SEM and were analyzed by one-way ANOVA and Newman-Keuls’s post hoc test (*p* < 0.05); *n* = 6 mice per group

Gut microbiota were also compared at the class by heatmap analysis (Fig. 4a). The bacteria were divided into a major class and a minor class (representing < 10% of the total proportion). *Clostridia, Bacteroidia,* and *Erysipelotrichi* constituted the major class of bacteria detected, whereas *Deltaproteobacteria, Deferribacteres_c, Gammaproteobacteria, Verrucomicrobiae*, and *Betaproteobacteria* constituted the minor class of bacteria. The proportion of *Bacteroidia* and *Gammaproteobacteria* were 50.3% and 4.1% in the DSS group, while *Clostridia* was decreased by 62.2% in the DSS group compared with the control group (Fig. 4b and c). In the SQE group, the proportion of *Clostridia* was more than two times higher, and the proportion of *Bacteroidia* and *Gammaproteobacteria* were significantly lower (83.6%), compared to the DSS group. Moreover, both *Bacteroidia* and *Gammaproteobacteria* almost recovered to control levels.

**Fig. 5:**
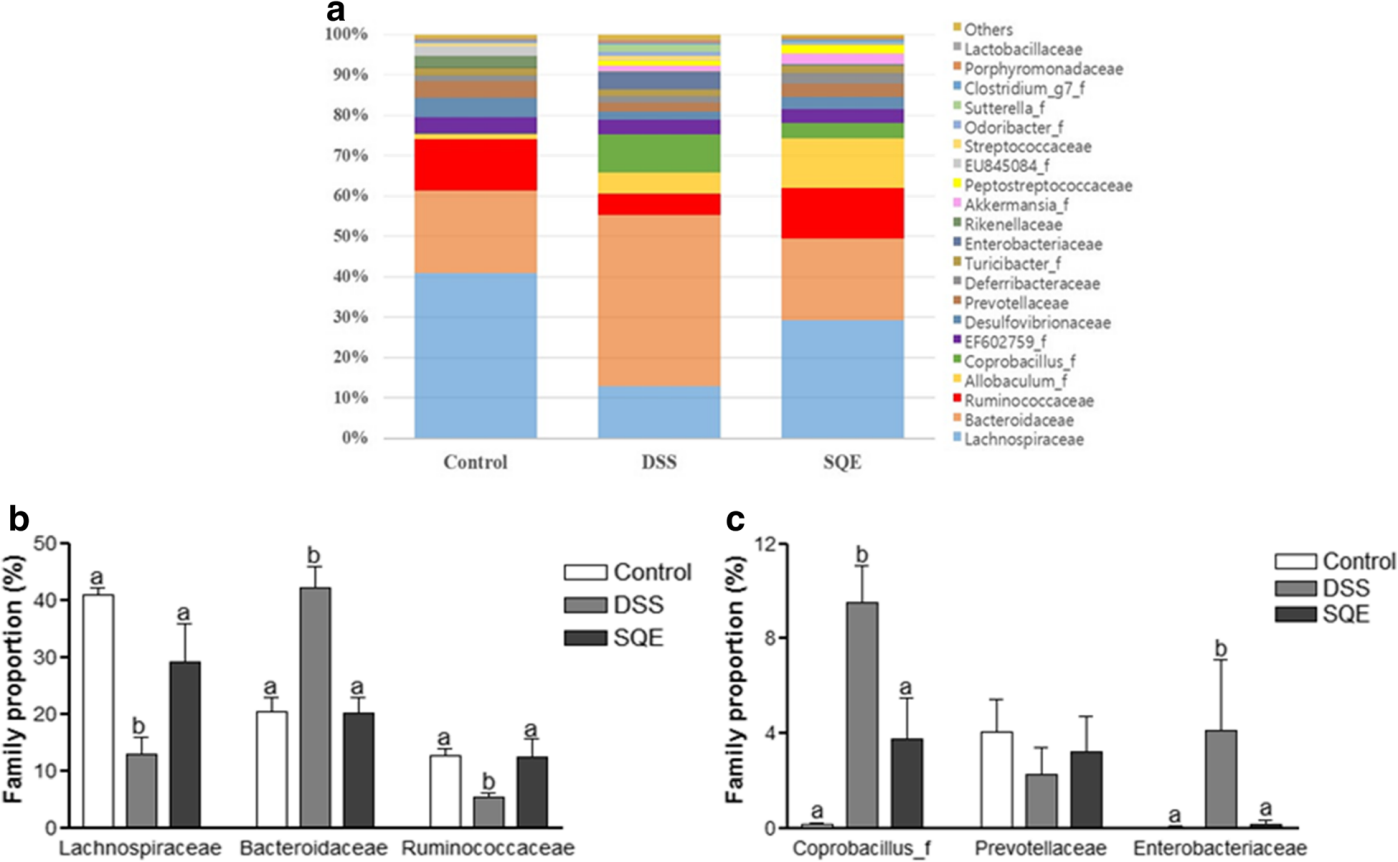
Composition of gut microbiota at the family level. **a** The composition of gut microbiota at the family level. **b** Relative abundance of the dominant family level in samples of control and DSS, SQE group. **c** Relative abundance of family level of minor proportion of bacteria in control, DSS, and SQE groups. Data shown are the means ± SEM and were analyzed by one-way ANOVA and Newman-Keuls’s post hoc test (*p* < 0.05); *n* = 6 mice per group

Colitis led to the dysbiosis of the intestinal microbiota in the DSS treated mice at the family level, similar to the observations made at the phylum and class levels. When differences in the microbiota at the family level were compared (Fig. 5a). *Lachnospiraceae, Bacteroidaceae*, and *Ruminococcaceae* were found to be the dominant bacteria in all three group. DSS treatment decreased the proportions of *Lachnospiraceae* (68.4%) and *Ruminococcaceae* (57.8%), and increased the proportion of *Bacteroidaceae* two-fold compared to the control group (Fig. 5b). With SQE supplementation, the proportion of all three bacteria returned to the levels of control group. When the bacteria were divided into a major family and a minor family of bacteria, *Lachnospiraceae, Bacteroidaceae,* and *Ruminococcaceae* constituted the are major family of bacteria, while *Coprobacillus*, *Prevotellaceae*, and *Enterobacteriaceae* constituted the minor family of bacteria (representing less than 10% of the total proporation). Among the minor bacteria, the proportion of *Coprobacillus*, and *Enterobacteriaceae* greatly increased following DSS treatment compared with the control group, and these increases were suppressed following SQE supplementation (Fig. 5c). In contrast, the abundance of *Prevotellaceae* and *Streptococcaceae* did not significantly differ among the three groups.

At the genus level, the proportion of *Clostridium, Bacteroides*, and *Enterobacter* significantly increased following DSS treatment compared to the control group, yet they decreased to control levels following SQE supplementation (Table 3). In contrast, the proportion of *Hungarella* and *Alistipes* significantly decreased following DSS treatment, while SQE supplementation tended to increase the proportion of these bacteria. At the species level, the proportion of *Bacteroides acidifaciens* (*p* < 0.001)*, Clostridium cocleatum* (*p* < 0.001), and unclassified *Bacteroides* (*p* < 0.01) were significantly higher in the DSS group compared to the control group. However, following SQE supplementation, the proportion of these bacteria decreased back to the proportions observed in the control group (Table 4). These results suggest that SQE supplementation attenuates intestinal bacteria dysbiosis by regulating the bacteria compositional changes in bacteria that are associated with DSS-induced colitis in mice.

**Table 3:** Composition of fecal microbiota in DSS – induced colitis mouse model^c^

Phylum	Genus	Control	DSS	SQE
*Firmicutes*	*Pseudoflavonifractor*	4.83 ± 0.82	2.71 ± 1.32	6.19 ± 3.93
	*Clostridium_g6*	0.17 ± 0.13 ^a^	9.49 ± 3.85 ^b^	3.75 ± 4.23 ^a^
	*Acetatifactor*	1.48 ± 1.42	1.31 ± 1.30	4.78 ± 3.55
	*Oscillibacter*	1.68 ± 0.75 ^a^	1.12 ± 0.76 ^a^	4.44 ± 2.68 ^b^
	*Hungatella*	3.56 ± 1.07 ^a^	0.82 ± 0.69 ^b^	1.50 ± 1.28 ^b^
	*Turicibacter*	1.72 ± 3.57	1.61 ± 1.73	1.73 ± 2.48
	*Clostridium_g21*	1.51 ± 0.59	0.75 ± 0.44	1.78 ± 1.44
	*Romboutsia*	0 ± 0	1.20 ± 2.93	2.09 ± 4.35
	*Lachnospiraceae_uc*	1.41 ± 0.47	0.25 ± 0.29	1.47 ± 1.75
	*Roseburia*	0.17 ± 0.08	0.11 ± 0.07	0.24 ± 0.17
*Bacteroidetes*	*Bacteroides*	20.23 ± 6.55 ^a^	41.64 ± 8.25 ^b^	20.10 ± 6.43 ^a^
	*Alloprevotella*	4.07 ± 3.37	2.20 ± 2.76	3.19 ± 3.61
	*Alistipes*	3.11 ± 1.12 ^a^	0.31 ± 0.23 ^b^	0.30 ± 0.16 ^b^
*Proteobacteria*	*Enterobacter*	0.02 ± 0.03 ^a^	3.94 ± 7.40 ^b^	0.04 ± 0.05 ^a^
	*Parasutterella*	0.01 ± 0.01	1.78 ± 3.55	0.04 ± 0.06
*Deferribacteres*	*Mucispirillum*	1.38 ± 1.66	1.64 ± 1.56	2.72 ± 3.00

^c^Cut-off: 1.0

Values are mean ± SD

Significantly different by one – way ANOVA and Newman-Keuls’s post hoc test among the three groups (*p* < 0.05); *n* = 6 mice per group

^ab^For a given column, data not sharing a common superscript letter significantly differ

**Table 4:** Species level bacteria proportion

Group Species	Control	DSS	SQE
*Bacteroides acidifaciens*	0.48 ± 0.41 ^a^	18.54 ± 9.75 ^b^	9.53 ± 6.55 ^a^
*Bacteroides sartorii*	7.03 ± 10.51	6.22 ± 8.79	0.19 ± 0.19
*Clostridium cocleatum*	0.17 ± 0.13 ^a^	9.43 ± 3.84 ^b^	3.73 ± 4.21 ^a^
*Mucispirillum schaedleri*	1.38 ± 1.66	1.64 ± 1.56	2.72 ± 2.98
*Enterobacter xiangfangensis*	0.01 ± 0.02	3.38 ± 6.37	0.04 ± 0.05
*Akkermansia muciniphila*	0 ± 0	1.24 ± 1.84	2.51 ± 4.24
*Romboutsia ilealis*	0 ± 0	1.17 ± 2.87	2.08 ± 4.32
*Lachnospiraceae_uc_s*	1.41 ± 0.47	0.25 ± 0.29	1.47 ± 1.75
*Bacteroides_uc*	0.30 ± 0.09 ^a^	1.85 ± 1.18 ^b^	0.73 ± 0.38 ^a^
*Butyricimonas virosa*	0.45 ± 0.17	0.47 ± 0.30	0.38 ± 0.19
*Ruminococcaceae_uc_s*	0.54 ± 0.49	0.07 ± 0.06	0.27 ± 0.23
*Lactococcus lactis subsp*	0.41 ± 0.32	0.23 ± 0.19	0.15 ± 0.11

Values are mean ± SD

Significantly different by one – way ANOVA and Newman-Keuls’s post hoc test among the three groups (*p* < 0.05); *n* = 6 mice per group

^ab^For a given column, data not sharing a common superscript letter significantly differ

## Discussion

The human gut contains a large population of diverse and complex enteric microbiota. Tremendous changes in the diversity and composition of this community, as well as the metabolic function of the gut microbiota, have been related to IBD [27, 28]. In particular, gut microbiota have been identified as a critical factor in IBD. Correspondingly, short-term antibiotic treatment for IBD patients have been used to suppress intestinal inflammation [29, 30]. Using murine model in gut microbiota study has been allowed functional and metabolic research on host-microbe interactions, and has brought more insights into the pathological mechanisms of IBD [31]. In colitis mouse model, the major gut microbiota shifted and gut bacterial diversity was reduced similar to those found in human IBD [32, 33].

Previously, it was reported that SQE treatment modulated the levels of proinflammatory markers, while also regulated the activation of nuclear factor κB and oxidative stress, in animal models of DSS-induced colitis [17, 34]. In the present study, the goal was to understand the effect of SQE on dysbiosis of microbiota in DSS-induced colitis. Therefore, overall differences in the microbial community, as well as modifications of microbiota composition after SQE treatment were investigated by using barcoded pyrosequencing of the 16S rRNA gene. The results obtained demonstrate that the microbial community profiles of the experimental groups examined were altered by DSS treatment, and dysbiosis of gut microbiota was improved with SQE supplementation.

In animal models of IBD, DAI value and colonic length are key indicators for evaluating the severity of colitis [35, 36]. Consistent with the results of a previous study [17], SQE supplementation attenuated the severity of colitis by lowering the DAI value and extending the length of the colon. In contrast, changes in the colon epithelium and higher DAI values characterized in the DSS group compared with the control group,

Modification to the composition of a microbial community may involve changes in diversity and in bacterial metabolism. Furthermore, an imbalance between obligate anaerobic bacteria and facultative anaerobic bacteria can occur, and this is related to the inflammation process [37, 38]. For example, Ott et al. reported that a microbial shift due to an increased in gram-negative bacteria accompanied a reduction in bacterial diversity in IBD patients, and this led to abnormalities in the inflammatory process [39]. In the present study, the analysis of various alpha diversity indices indicated that a reduction in bacterial diversity occurred in the DSS group compared to the control and SQE groups. In addition, the gut microbial communities of the DSS group were characterized by a clustered distance to the control group. The latter result is consistent with the results of previous studies where microbial divergence manifested as relative abundance shift in cases of IBD [39, 40]. However, in the present study, SQE supplementation recovered the bacterial diversity of the gut and greater clustering of the gut microbial communities close to the control group was observed compared to the DSS group. Taken together, these results suggest that SQE may help the gut microbiota to maintain their composition, community, microbial evenness, and richness.

Interactions between gut microbiota and the host immune system play an important role in the development of a host’s immune system [41]. Generally, the composition of gut microbiota remains stable during adulthood, and it can undergo dynamic changes in response to environmental stresses or diet. Such alterations in composition may influence health or disease risk [42]. The taxonomic compositions of the gut microbiota in humans is similar to that observed in mice at the phylum level [43]. Dysbiosis in patients with IBD has been characterized as an increase in the ratio of *Bacteroidetes*/*Firmicutes* [28, 44]. In the present study, the ratio of *Bacteroidetes*/*Firmicutes* was significantly higher in the DSS group than in the control group, whereas this ratio in the SQE group was similar to that of the control group. It was also observed that the proportion of *Firmicutes* was significantly decreased following DSS treatment, yet the proportion recovered to control levels following SQE supplementation. The *Firmicutes* phylum modulates the pH of the colonic and inhibits the growth of pathogens by metabolizing short-chain fatty acids (SCFAs) and producing butyrate in the intestinal mucosa. Butyrate is a key energy source for epithelial cells of the colon and it suppresses pro-inflammatory cytokines in the gut [45]. At the class level, an increase in *Bacteroidia* (phylum *Bacteroidetes*) and *Gammaproteobacteria*, as well as a reduction in *Clostridia* (phylum *Firmicutes*) was observed in the DSS group compared to the control group. *Gammaproteobacteria*, a bacteria that can induce acute intestinal inflammation, was also significantly increased in the DSS group, thereby indicating that changes in intestinal permeability and induction of chronic systemic inflammation had occurred [46]. However, these changes in the composition of the microbial community were reduced in the SQE group compared to the DSS group, which suggested that SQE was able to regulate a gut microbial community by modulating gut inflammation.

In patients and experimental animal models with IBD, the relative abundance of *Lachnospiraceae* was reduced and the proportion of the *Bacteroidaceae* is relatively increased [47]. *Lachnospiraceae* plays an important role in fermenting SCFAs that derived from carbohydrates [48]. Another bacteria, *Ruminococcaceae* performs the first step in carbohydrate metabolism where hydrogen is consumed to butyrate. Microbial metabolisms of SCFAs is associated with gut motility and intestinal transit time, as well as with the function of histone deacetylases and the nervous system [49]. In the present study, the compositional abundances of *Lachnospiraceae*, *Bacteroidaceae*, and *Ruminococcaceae*, which mediate SCFA metabolism, were altered in mice of the DSS group. In contrast, microbial dysbiosis was improved in the SQE group compared with the DSS group. Previously, it was reported that SQE facilitated gut motility in the DSS-induced colitis mouse model [34], and this explains the role of SQE in the metabolisms of SCFAs and microbial composition related to intestinal function. *Enterobacteriaceae* (genus *Enterobacter*)*,* obligate anaerobic bacteria for the metabolism of high energy nutrients, is present in greater number during inflammation [50]. In the present study, an increase in the proportion of *Enterobacteriaceae* was consistently detected in the DSS group compared with the control group, and this increase was blocked with administration of SQE.

As presented above, a strong connection between gut microbiota and the intestinal immune system has been observed. Among the various microbacteria, *Clostridium* (species *Clostridium cocleatum*) and *Bacteroides* (species *Bacteroides acidifaciens*, *Bacteroides_uc*) have been reported to induce the emission of regulatory T cells and to reduce intestinal inflammation [51]. In the present study, higher proportion of the *Clostridium* and *Bacteroides* were detected in the DSS group compared with to the control group, and these increase suggest that prevention of intestinal inflammation by specific groups of commensal obligate anaerobic bacteria may mediate direct protective effects for pathogens. Furthermore, the balance of microbial composition of species affects the bile acid metabolism in the colon. In particular, *Enterobacter*, *Bacteroides*, and *Clostridium* absorb dietary fats, facilitate lipid absorption, and maintain intestinal barrier function [52]. Consequently, dysbiosis resulting from intestinal inflammation can affect the function of bacteria and the other metabolic processes.

Many polyphenols contribute to important biological activities, including antioxidant, anticarcinogenic, and antimicrobial activities that are associated with pathological disease processes [53, 54]. In addition, most polyphenols are consumed and ingested, after being metabolized by gut microbiota, which leads to greater biological activity and increased bioavailability compared with their predecessors [55]. Furthermore, polyphenol intake may have a direct impact on the composition of gut microbiota and the functionality and the growth of certain bacterial species. For example, in the presence of phenolic compounds, the *Firmicutes*/*Bacteroidetes* ratio in the microbiota of obese individuals was found to be altered, and polyphenol-rich grape seed extract has been found to contain a higher proportion of *Lactobacillus*/*Enterococcus* bacteria [56, 57]. Several studies have also shown that natural herbs and polyphenols help to improve intestinal inflammation in colitis model [58]. SQE has shown beneficial effects on colitis in previous studies. Moreover, the bioactive component of SQE, tricin and *p*-coumaric acid, have exhibited antioxidant, anti-inflammatory, and anticancer effects which remain to be investigated in relation to gut microbiota [17, 18, 20, 34].

The identification of host and microbial interactions in IBD patients, as well as a greater understanding of the role of the microbiome and the changes in its composition that occur in the disease states of IBD, should lead to the development of highly effective and nontoxic targeted interventions to correct underlying abnormalities and induce sustained therapeutic responses. Currently, broad spectrum antibiotics, probiotics, and prebiotics are used to prevent and treat IBD [59]. The present results suggest a possible role for SQE and its various of polyphenols in the clinical treatments of IBD via regulation of gut microbiota dysbiosis and diversity. Moreover, the use of SQE would represent a natural therapeutic strategy for IBD patients. However, a clinical intervention trial is needed to confirm the present results in IBD patients, while additional research is needed to understand the relationship between dietary polyphenols and gut microbiota.

## Conclusions

The present study we demonstrated that DSS-induced colitis changed the diversity of the intestinal microbial composition and diversity led to an increase of inflammation in colon. However, when SQE was administered prior to the induction of colitis by DSS, microbial dysbiosis was reduced. These results increase our understanding of the important role that gut microbacteria have in maintaining intestinal homeostasis, and they also support the natural therapeutic potential of SQE for modulating dysbiosis in IBD.

## Abbreviations

CD: Crohn’s disease
DAI: Disease activity index
DSS: Dextran sulfate sodium
IBD: Inflammatory bowel disease
OTUs: Operational taxonomic units
PCoA: Principal coordinate analysis
SQE: *Sasa quelpaertensis* leaf extract
SSZ: Sulfasalazine
UC: Ulcerative colitis
